# Efficacy and safety of upadacitinib in treating immune drift: a single-center, prospective study in China

**DOI:** 10.3389/fimmu.2026.1842140

**Published:** 2026-07-06

**Authors:** Wanyan Xiang, Jiarong Lu, Siqi Zhang, Wenjun Zheng, Qiuju Li

**Affiliations:** Department of Dermatology, The First Affiliated Hospital of Guangxi Medical University, Nanning, China

**Keywords:** atopic dematitis, psoriasis, immune drift, psoriais, treatment, upadacitinib

## Abstract

**Introduction:**

With the widespread use of biologic agents in dermatology, immune drift has emerged as a growing clinical challenge. Although several case reports suggest the therapeutic potential of JAK inhibitors, clinical evidence for upadacitinib—a highly selective JAK1 inhibitor—remains limited.

**Methods:**

A prospective study evaluated the efficacy and safety of upadacitinib in patients with immune drift at the Department of Dermatology, First Affiliated Hospital of Guangxi Medical University, from December 2024 to September 2025.

**Results:**

Eleven patients exhibiting immunological drift (including eight with PSO-AD and three with AD-PSO) were recruited for this investigation, with a mean age of 37.4 ± 18.8 years. Following four weeks of treatment, their IGA 0/1 response rates reached 62.5%. Their EASI scores decreased markedly from 18.0 (12.4–24.4) to 2.5 (0.52–13.1) (*p* = 0.0059) with EASI-75 and EASI-90 response rates of 63.64% and 45.5%, respectively. Their PASI scores declined significantly from 9.4 (7.4–10.2) to 2.8 (1.85–4.05) (*p* = 0.001), with PASI-75 and PASI-90 response rates of 45.45% and 18.18%, respectively. Their SCORAD 50 and SCORAD 75 response rates were 72.73% and 36.36%, respectively. The patients’ quality of life improved markedly, as evidenced by a reduction in their DLQI scores from 21.8 ± 1.75 to 9.3 ± 8.92 (*p* = 0.0012) and 54.55% of the patients showing an NRS improvement of ≥4 points. The subgroup analysis revealed an average percentage improvement in EASI score of 72.33% in the PSO-AD cohort and 63.38% in the AD-PSO cohort. No baseline factors were found to be statistically significant predictors of treatment response. In terms of safety, no severe adverse events were documented, and only one patient (9.1%) experienced folliculitis.

**Conclusion:**

Our findings suggest that upadacitinib is effective and safe in managing immune drift, with rapid improvements in disease control and quality of life.

## Introduction

In dermatology, immune drift has gained increasing recognition in patients with psoriasis (PSO) or atopic dermatitis (AD) undergoing prolonged treatment with biological agents. However, its exact incidence remains unclear. In general AD populations, the prevalence of coexisting psoriasis ranges from 0.3% to 12.6% (1), but the occurrence of PSO-like lesions specifically induced by AD biologics (e.g., dupilumab) has been reported in approximately 1-5% of treated patients. Conversely, eczema-like rashes in PSO patients receiving IL-17 or IL-23 inhibitors are less frequent, estimated at <1-2% (2, 3). This presents as PSO-like rashes in AD patients treated with interleukin-4/13 (IL-4/13) inhibitors (PSO-AD) (2), and eczema-like rashes in PSO patients treated with Tumor Necrosis Factor-α(TNF-α), IL-17A, IL-12/23, or IL-23 inhibitors (AD-PSO) (4). This phenomenon could be mechanistically linked to the disruption of the Th1/Th2/Th17 immunological axis and the continual restructuring of the cytokine network. Conventional therapies (e.g., cyclosporine or combinatory treatments) frequently lack efficacy and are prone to causing side effects or recurrences.

Upadacitinib, a highly selective oral JAK1 inhibitor, laterally inhibits JAK signaling by suppressing both Th2 and Th17 pathways (5). Theoretically, iit may simultaneously regulate both disease phenotypes. Despite several case reports supporting its use, systematic clinical data on its safety and efficacy remain scarce.

Therefore, this prospective observational study aimed to systematically evaluate the efficacy and safety of upadacitinib in Chinese patients with immune drift, addressing a critical gap in clinical evidence.

## Methods

This was a single-center, prospective observational cohort study. Patients for whom the treating physician had already decided to initiate oral upadacitinib for immune drift complicating AD or PSO were enrolled. Investigators did not intervene in treatment decisions, assign a control group, or mandate specific dosing regimens. The research team systematically collected and assessed data according to a prospective protocol. The study was approved by the Ethics Committee of the First Affiliated Hospital of Guangxi Medical University (Approval No. 2026-E0284) and conducted in accordance with the Declaration of Helsinki. Written informed consent was obtained from all adult participants and from parents/legal guardians of minors.

All patients met the following four criteria: 1. Baseline stability: Moderate-to-severe AD or PSO controlled (IGA 0–2) for ≥3 months on a stable biologic. 2. New distinct lesions: After biologic initiation, lesions atypical for the original disease: PSO-AD: well-demarcated, scaly plaques (psoriasis-like). AD-PSO: ill-defined, pruritic, eczematous patches. 3. Conventional treatment failure: <50% improvement after ≥4 weeks of appropriate topical/systemic therapy (corticosteroids, calcineurin inhibitors, cyclosporine, or oral corticosteroids). 4. Exclusion of differentials: Contact dermatitis, infection, drug eruption ruled out. In ambiguous cases (3/11, 27.3%), biopsy confirmed histopathology (parakeratosis with neutrophils for PSO-like; spongiosis with lymphocytes for eczema-like).

The inclusion criteria for immune drift were as follows: (1) a recorded history of AD or PSO; and (2) the patient readily accepted upadacitinib treatment and signed the informed consent form. The exclusion criteria were as follows: (1) urgent medical conditions (including active infection, malignant neoplasm, severe hepatic or renal impairment); (2) coexisting autoimmune disorders (such as systemic lupus erythematosus or rheumatoid arthritis); and (3) pregnant or breastfeeding women.

Eligible patients were continuously recruited from the outpatient and inpatient departments of the Department of Dermatology at the First Affiliated Hospital of Guangxi Medical University between September 2024 and December 2025. All enrolled patients were those for whom the clinician had independently determined to discontinue the existing biologic agent and initiate oral upadacitinib as monotherapy for the management of immune drift. Therefore, all participants in this observational cohort received oral upadacitinib without continuation of the prior biologic. The treatment regimen (e.g., 15 mg once daily) was determined by the treating physician according to clinical judgment and routine practice. Upadacitinib was not added to the existing biologic; rather, it replaced the previous biologic therapy. The primary efficacy was the proportion of patients achieving an Investigator’s Global Assessment (IGA) 0/1 at Week 4. Key secondary objectives included: (1) the proportion of patients who achieved at least a 75%, 90%, or 100% reduction in the Eczema Area and Severity Index (EASI) score from baseline (EASI-75, EASI-90, and EASI-100 response rates, respectively); (2) the proportion of patients who achieved at least a 75% or 90% reduction in the Psoriasis Area and Severity Index (PASI) score from baseline (PASI-75 and PASI-90 response rates, respectively); (3) the proportion of patients with a reduction of ≥4 points in the Peak Pruritus Numerical Rating Scale (NRS); (4) The change from baseline in the Dermatology Life Quality Index (DLQI). Safety was assessed by monitoring and recording all adverse events throughout the study period.

Data analysis was performed using R 4.4. The Shapiro-Wilk test was employed to assess the normality of the continuous variables. When the data followed a normal distribution, they were presented as the mean ± standard deviation ( $\bar{x}$ ± s). Comparisons between the groups were conducted using the paired t-test. The median interquartile range [M(Q1-Q3)] was used to describe the non-normally distributed data, and the Wilcoxon signed-rank test was used to perform intergroup comparisons. The categorical variables were summarized as counts (percentages), and the group comparisons were carried out using either the chi-square (χ²) test or Fisher’s exact test where appropriate. The primary endpoint (defined as the IGA 0/1 response rate) was reported as a percentage value. As for the secondary endpoints (EASI, PASI, NRS, and DLQI), the statistical significance of any changes that occurred from baseline to Week 4 was evaluated via difference analysis. A two-tailed P-value < 0.05 constituted a statistically significant difference.

Of note, this was a non-interventional, prospective observational study: the decision to prescribe upadacitinib was made by clinicians independently of the research protocol, and all patients received upadacitinib monotherapy. There was no control group or randomization.

## Results

### Patient characteristics

Eleven patients were included, consisting of nine females and two males, with a mean age of 37.4 ± 18.8 years. Among them, eight patients (72.7%) were classified as PSO-AD, and three (27.3%) as AD-PSO. In the PSO-AD subgroup, one had a family history of AD, and two had allergic rhinitis. Only one patient was diagnosed with psoriatic arthritis. Laboratory analyses revealed elevated serum IgE levels in seven patients (88%, 7/8) and increased eosinophil counts in one patient. In the AD-PSO subgroup, one had a family history of AD, but none had a family history of psoriasis. IgE levels increased in two cases, despite their eosinophil counts remaining within the normal range. All patients had been on a stable biologic regimen for at least three months before the onset of immune drift. After the emergence of immune drift (i.e., new, morphologically distinct lesions), all patients exhibited poor response to conventional therapies appropriate for the new phenotype, including topical agents (potent corticosteroids or tacrolimus ointment) and/or systemic therapies (cyclosporine or oral corticosteroids), necessitating a change in treatment strategy (**Table 1**).

**Table 1:** Clinical characteristics, adverse effects and response to upadacitinib of histologically confirmed cases of overlapped psoriasis and atopic dermatitis.

Patient	1	2	3	4	5	6	7	8	9	10	11
Age (years)	20	37	45	39	52	65	50	38	6	54	5
Sex	M	M	M	M	M	F	M	F	F	M	F
Family history of atopic disease	No	No	Yes	No	No	No	No	No	Yes	No	No
Duration of primary disease (years)	>10	2	4	4	36	1	>10	1	5	7	5
Atopic disease comorbidities	No	No	allergic rhinitis	No	No	allergic rhinitis	No	No	Urticaria	Urticaria, Allergen positive	Allergen positive
Psoriasis type	PV	PV	PV	PV	PSA	PV	PV	PV	PV	PV	PP
Psoriatic arthritis	No	No	No	No	Yes	No	No	No	No	No	No
Treatments before immune drift	secu	secu	ADA, secu	Ixek	secu/ust	secu	secu	Til	AH, dupi	AH, CsA, dupi, Abro	AH, CsA, dupi
Pattern	PSO-AD	PSO-AD	PSO-AD	PSO-AD	PSO-AD	PSO-AD	PSO-AD	PSO-AD	AD-PSO	AD-PSO	AD-PSO
Baseline IgE	1595.2	154.7	258.8	125.6	176.5	5000	1849.3	21.6	277.9	31.8	252.6
Baseline EOS	0.25	0.05	0.36	0.19	0.02	–	0.63	0.53	0.8	0.5	0.075
Baseline EASI	16.8	8.6	11.2	8	16	24.5	24.1	40.6	19.2	8	72
Baseline PASI	9.9	5	10.4	7.8	10	7	14.9	9.4	4	8.6	35.8
Baseline IGA	3	4	4	4	3	3	4	–	4	3	–
Baseline DLQI	24	21	21	21	22	21	22	22	23	18	24
Baseline NRS	6	6	8	7	7	6	8	9	7	7	10
Week4_EASI	3	0.6	0	0	8	0.5	14.8	22.5	0	2	61.1
Week4_PASI	4.4	0.5	3.2	3.7	4.7	2.4	2.8	1.9	0	1.8	9.9
Week4_IGA	1	1	1	–	3	1	3	–	1	2	–
Week4_DLQI	1	5	0	–	21	5	13	18	2	4	24
Week4_NRS	3	3	0	–	6	2	6	8	1	2	9
Upacitinib dose (mg)	15 qd	15 qd	15 qd	15 qd	15 qd	15 qd	15 qd	15 qd	15 qd	15 qd	15 qd
Treatment duration (m)	9	–	6	3	2	2	6	6	9	12	2
Primary disease outcomes	Improved	lost to follow-up	Improved	poor	Improved	Improved	Improved	Improved	Improved	Improved	Improved
Post-drift disease outcomes	Improved	lost to follow-up	Improved	Improved	poor	Improved	Improved	Improved	Improved	Improved	poor
Adverse	–	–	–	–	–	–	–	–	–	–	Folliculitis rash

PV: Psoriasis Vulgaris, PsA: Psoriatic Arthritis, PP: Pustular Psoriasis, secu: secukinumab, ada: Adakimumab, Ixek: Ixekizumab, ust: Ustekinumab, Til: Tildrakizumab, AH: Antihistamines, CsA: Cyclosporine, dupi: Dupilumab, abro: Abrocitinib.

### Upadacitinib improves EASI, IGA, DLQI and NRS in patients with immune drift

All the patients demonstrated a baseline IGA ranging from 3 to 5. After 4 weeks of treatment with upadacitinib (15mg q.d or q.o.d.), significant clinical improvements were observed. The IGA 0/1 response rate reached 62.5%. The EASI score decreased from 18.0 (12.4-24.4) at baseline to 2.5 (0.52-13.1) (P = 0.0059). The EASI-75 and EASI-90 were 63.6% and 45.5%, respectively. The PSO-AD group exhibited an average percentage improvement in EASI score of 72.33%, while the AD-PSO group showed an improvement of 63.38%. Male and female patients demonstrated improvement rates of 73.13% and 64.42%, respectively. This indicates that the efficacy trend was similar across different subgroups.

**Figure 1:**
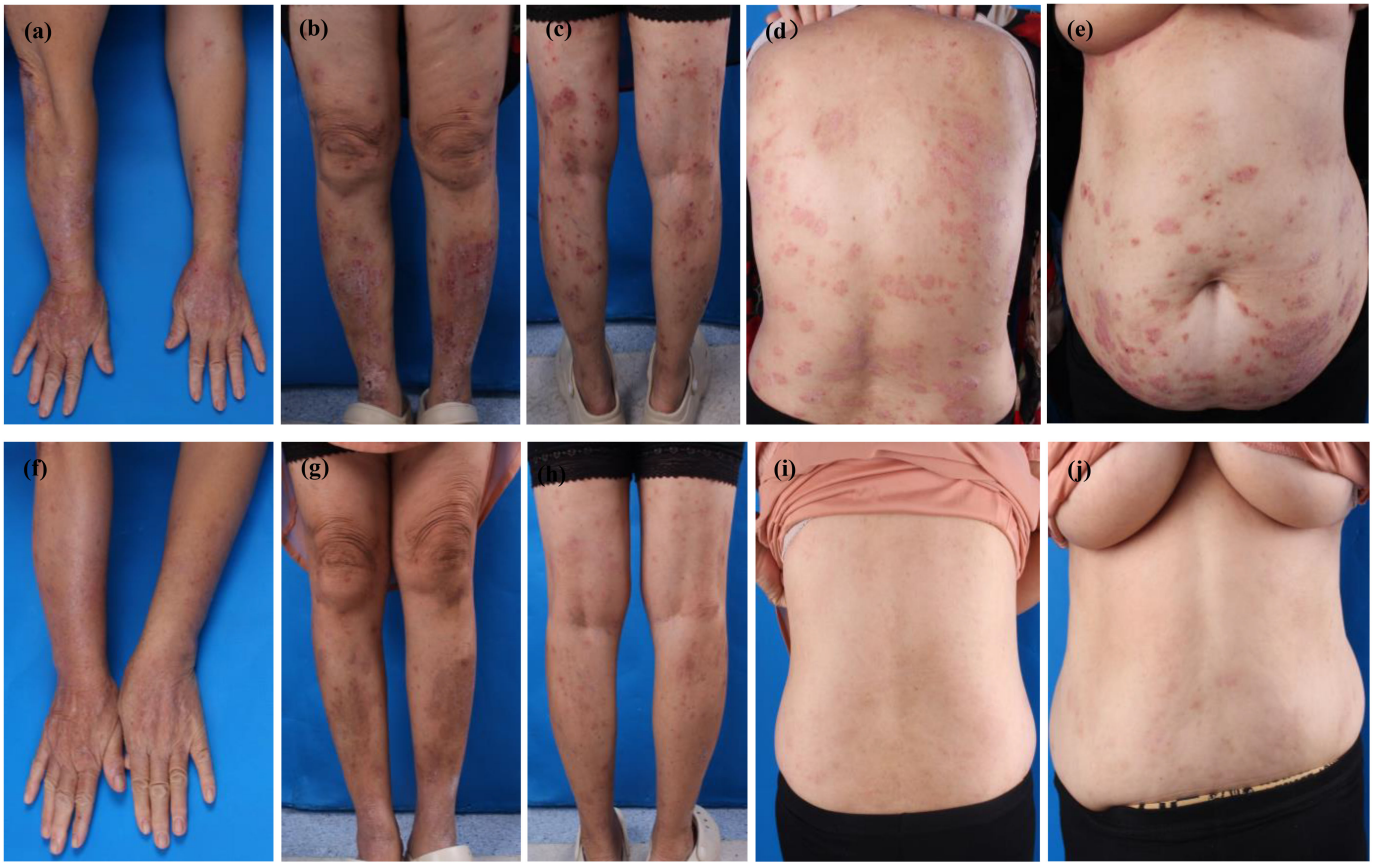
Clinical response to upadacitinib in a patient with PSO-AD. **(a–e)**, before using upadatinib. **(f–i)**, after 4 weeks of upadatinib.

**Figure 2:**
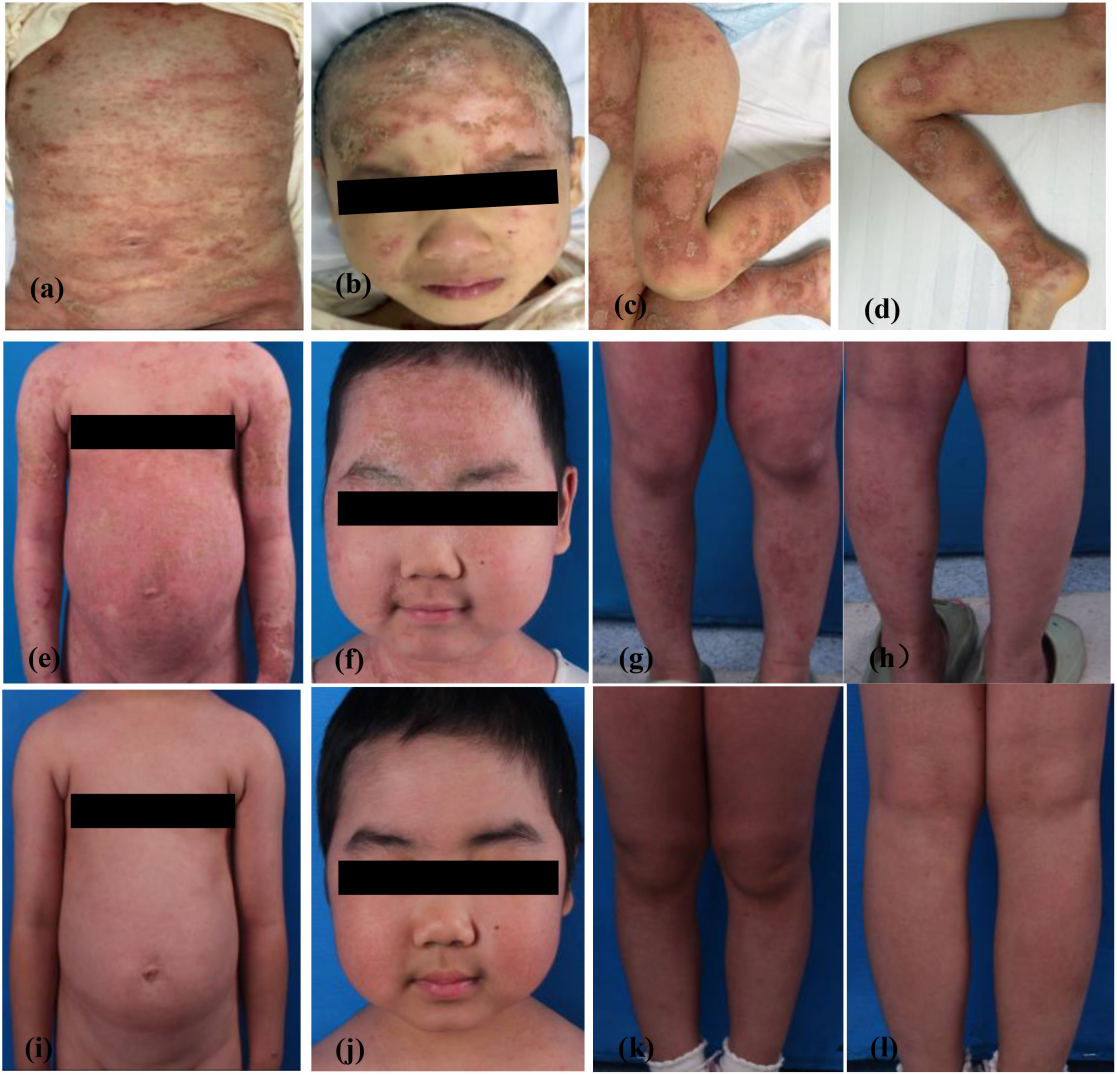
Clinical response to upadacitinib in a patient with AD-PSO. **(a–d)** before using upadatinib. **(e–h)** after 4 weeks of upadatinib. **(i–l)** after 8 weeks of upadatinib.

**Figure 3:**
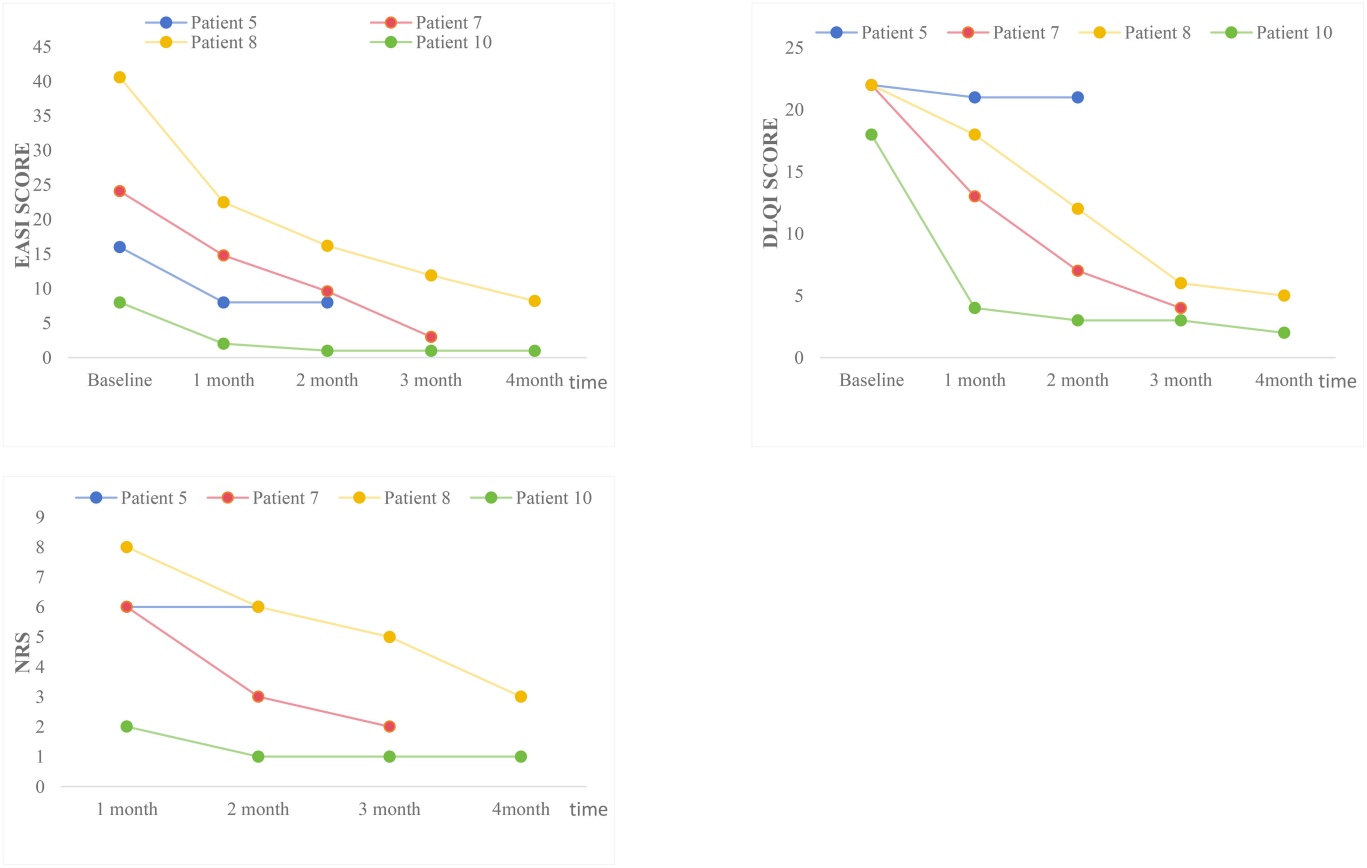
Follow-up changes in EASI **(a)**, DLQI **(b)**, and NRS **(c)** scores in four patients.

The DLQI score improved significantly from 21.8 ± 1.75 to 9.3 ± 8.92 (P = 0.0012), and the pp-NRS score decreased from 7.4 ± 1.35 to 4.0 ± 3.06 (P = 0.0014). The percentage of patients with an NRS ≥4 was 54.55%, indicating a substantial improvement in both quality of life and pruritus symptoms. These data indicate that upadacitinib not only ameliorates AD skin lesions but also rapidly alleviates pruritus and significantly enhances quality of life in patients with immune drift (**Figures 1**–**3**).

### Upadacitinib improves PASI in patients with immune drift

Concurrently, the PASI score declined from 9.4 (7.4-10.2) to 2.8 (1.85-4.05) (P = 0.001), with PASI-75 and PASI-90 achieved by 45.45% and 18.18% of patients, respectively. Post-treatment PASI scores stabilized around 2–3 points, with approximately 73% of patients experienced no recurrence of psoriasis. Upadacitinib treatment resulted in rapid and simultaneous reduction of both AD and PSO severity scores, demonstrating its dual efficacy in immune drift.

### No significant correlation between treatment effectiveness

An evaluation of the efficacy predictors revealed no significant correlation between treatment effectiveness (defined as an ≥50% improvement in EASI score), age (*p* = 0.604), gender (*p* = 0.500), family history of atopic disease (*p* = 1.000), baseline IgE level (*p* = 0.833), baseline eosinophil count (*p* = 0.715), or disease pattern (*p* = 1.000). These findings suggest that upadacitinib is broadly effective across diverse patient subgroups, regardless of baseline clinical or laboratory characteristics. Given the small sample size (n=11), these exploratory analyses should be interpreted as descriptive only; the lack of statistically significant associations does not exclude true clinical predictors, and the statistical power for detecting such associations is limited.

### Upadacitinib shows sustained disease control during follow−up in most patients with immune drift

During the follow-up period, PSO(80%) and AD(90%) activity remained well-controlled in the majority of patients. In four patients with extended follow-up data beyond two months, EASI scores, DLQI and NRS continued to show sustained improvement (**Figures 1a–c**).

At the most recent follow-up evaluation, 1 patient was lost to follow-up and 1 patient in PSO-AD group discontinued upadatinib due to the development of PSO skin plugs and was switched back to ixekizumab. The remaining patients did not experience significant AD or recurrence of PSO. In the PSO-AD and AD-PSO groups, one patient in each group experienced immune drift with suboptimal disease control. Excluding those lost to follow-up or switched back to biologic therapy, the remaining nine patients continued upadacitinib treatment, despite unsatisfactory efficacy in one patient from each of the two groups.

Overall, upadacitinib demonstrated sustained efficacy and was well-accepted in most patients with immune drift, highlighting its potential as a long-term management strategy. While these observations suggest potential for sustained efficacy, the follow−up period was limited (up to 12 months in some patients), and larger studies with longer follow−up are needed to confirm long−term disease control and relapse rates.

### Upadacitinib has great safety profile in treating patients with immune drift

Treatment with upadacitinib was generally well-tolerated. Only one patient(9.1%) experienced a mild to moderate folliculitis rash, which did not necessitate treatment discontinuation. No serious adverse events, including infections, hepatic or renal dysfunction, or hematological abnormalities, were reported throughout the study period.,It demonstrated that upadacitinib was well tolerated in this cohort.

## Discussion

With the widespread adoption of targeted biologic agents in dermatology, a thought-provoking phenomenon known as immunological drift has emerged in clinical practice. Although its precise etiology remains incompletely understood, it is widely hypothesized to result from the dynamic imbalance of the immunological axis. For instance, the use of IL-17 inhibitors in PSO may relieve Th2 pathway inhibition, leading to the development of an AD-like phenotype. Conversely, the use of dupilumab in AD may disrupt the existing immune equilibrium by blocking IL-4/IL-13, thus potentially manifesting as a psoriasis-like rash dominated by the Th17/IL-23 axis (6, 7). Therefore, an ideal therapeutic strategy should possess upstream, broad-spectrum immunomodulatory capability to correct multi-axis imbalance synergistically.

Upadacitinib, a highly selective oral JAK1 inhibitor, showed efficacy in this study that aligns with its known mechanism of action. The JAK-STAT pathway serves as a common downstream hub for signal transduction of multiple cytokines, including interleukin (IL)-4, IL-13 (key Th2 axis cytokines), IL-23, and IL-17 (key Th17 axis cytokines). By inhibiting JAK1, upadacitinib can simultaneously and upstream block​ both the Th2 pathway driving AD and the Th17 pathway driving psoriasis.^3^ This “multi-point synergy” at the mechanistic level theoretically equips it to address the mixed lesions with both AD and psoriasis features resulting from immune axis drift. Our observations that upadacitinib treatment not only rapidly improved AD features but also attenuated psoriasis-like lesions provide indirect clinical evidence supporting this mechanistic hypothesis. Compared to biologics targeting a single pathway, this broad-spectrum regulatory characteristic of upadacitinib may offer a superior mechanism-driven treatment choice for managing the complex state of “immune drift,” which involves dynamic changes in multiple pathways.

Recognizing the clinical challenge of identifying immune drift patients, a recent study by Müller et al. developed and validated the first clinical algorithm (“Flip-Flop” score) to identify phenotypic switches between AD and psoriasis, achieving an overall accuracy of 89.7% in a cohort of 300 Caucasian patients (8). Although their algorithm relies on clinical features rather than biomarkers, it provides a structured approach to diagnosing this phenomenon. Notably, among their 13 FF patients, the majority (61.5%) experienced phenotypic switches during biologic therapy, including under IL-17A inhibitors (secukinumab) and IL-12/23 inhibitors (ustekinumab), consistent with our observations in AD-PSO patients. This independent validation of immune drift as a clinically recognizable entity supports the external validity of our findings, while also highlighting the ongoing need for objective diagnostic tools, particularly for non-Caucasian populations.

The results of this study are consistent with previous reports from key clinical trials (e.g., AD Up, PSO Achieve 1&2) on the efficacy and safety of upadacitinib in AD and psoriasis,^6,7^ further confirming the solid efficacy of this drug across different inflammatory skin diseases. More importantly, this study extends its application scenario to the special complex phenotypes emerging after conventional biologic therapy, preliminarily validating its practical value in such clinical dilemmas. However, the observation period of this study was 4 weeks, primarily assessing short-term efficacy and safety. The efficacy of long-term upadacitinib use (especially beyond 16 weeks or even one year) in maintaining remission of “immune drift,” its impact on disease phenotype stability, and long-term safety data still require confirmation through studies with larger samples and extended follow-up.

Our study systematically evaluated the efficacy and safety of upadacitinib in Chinese patients with confirmed immune drift. Over 50% of participants achieved an NRS improvement of ≥4 points within four weeks of treatment, indicating that JAK inhibitors can directly target pruritogenic cytokines, including IL-4, IL-13, and IL-31. This rapid anti-pruritic effect notably reduces patient discomfort and may contribute to enhanced treatment adherence. Moreover, clinically and statistically significant improvements were observed across key secondary endpoints: EASI-75 (63.64%), PASI-75 (45.45%), and DLQI (mean reduction of 12.5 points), collectively reflecting substantial gains in disease control and quality of life.

In particular, it is noteworthy that upadacitinib has been approved in China for the treatment of psoriatic arthritis and moderate-to-severe atopic dermatitis, but not yet for psoriasis vulgaris. However, this study demonstrated that upadacitinib treatment significantly reduced both PASI and EASI scores and maintained psoriatic lesions in a stable condition, with PASI scores remaining around 2–3 and no significant recurrence observed. These findings suggest that upadacitinib may effectively control both atopic dermatitis-like and psoriasis-like clinical manifestations through upstream, broad-spectrum immunomodulatory mechanisms, thereby offering a mechanistically aligned therapeutic option for patients exhibiting immune drift with overlapping disease features.

The subgroup analyses revealed that the efficacy trend of upadacitinib remained consistent irrespective of illness pattern (with a 72.33% improvement rate for PSO-AD and a 63.38% improvement rate for AD-PSO) or patient gender, thereby underscoring its extensive therapeutic potential.

Regarding safety, only one patient (9.1%) experienced a mild-to-moderate folliculitis rash, which did not require treatment discontinuation. No serious adverse events such as infections, hepatic/renal dysfunction, or hematologic abnormalities were reported. This safety profile aligns with existing evidence from large-scale clinical trials of upadacitinib in atopic dermatitis and psoriasis (9).

Existing literature includes case reports describing successful treatment of dupilumab-induced psoriasis with tralokinumab, an anti-IL-13 monoclonal antibody (10). This strategy involves switching to another AD-targeted biologic with a narrower mechanism of action to avoid exacerbating psoriatic features. In contrast, upadacitinib simultaneously modulates both inflammatory axes through upstream, broad-spectrum immunoregulation, which may represent a more advantageous approach for patients with complex, mixed AD-PSO phenotypes secondary to immune drift.

This study has the following limitations that should be considered when interpreting the results. First, it is a single-center, prospective observational study with a relatively small sample size (n=11), which may limit the statistical power and generalizability of the findings to different patient subgroups (e.g., different drift phenotypes, severity levels). Second, the lack of a randomized, double-blind, placebo-controlled design cannot completely rule out placebo effects and observer bias. Third, as a prospective observational study without a control group, the treatment effect observed cannot be definitively attributed to upadacitinib alone, and the findings should be interpreted within the context of standard clinical practice where this agent was uniformly chosen by clinicians. The lack of randomization and a control group is an inherent limitation of the observational design. Finally, although we used explicit clinical criteria, immune drift diagnosis relies on clinical judgment. Our prior work (11) employed a multidimensional approach (histopathology, IgE, cytokines) to improve accuracy, but without validated biomarkers, inter-physician variability remains inevitable. This limitation highlights the urgent need for objective diagnostic tools.

## Conclusion

In summary, the preliminary evidence from this prospective study suggests that for immune drift triggered by biologic therapy, characterized by the mutual transformation of AD and psoriasis features, upadacitinib is a treatment option capable of rapidly, effectively, and simultaneously improving lesions and core symptoms of both conditions, with good short-term tolerability. By inhibiting upstream JAK1, it achieves synergistic regulation of the key Th2 and Th17 signaling pathways, providing a powerful mechanism-driven therapeutic strategy for managing such complex clinical presentations involving multi-immune axis imbalance. The findings of this study support conducting larger-scale, longer-cycle research in the future to further establish the value of upadacitinib in managing the clinical challenge of immune drift.

## Data Availability

The original contributions presented in the study are included in the article/supplementary material. Further inquiries can be directed to the corresponding author.
